# OASIS modulates hypoxia pathway activity to regulate bone angiogenesis

**DOI:** 10.1038/srep16455

**Published:** 2015-11-12

**Authors:** Min Cui, Soshi Kanemoto, Xiang Cui, Masayuki Kaneko, Rie Asada, Koji Matsuhisa, Keiji Tanimoto, Yuki Yoshimoto, Chisa Shukunami, Kazunori Imaizumi

**Affiliations:** 1 Department of Biochemistry, Institute of Biomedical and Health Sciences, Hiroshima University, Hiroshima 734–8553, Japan; 2 Department of Radiation Medicine, Research Institute for Radiation Biology and Medicine, Hiroshima University, Hiroshima 734–8553, Japan; 3 Department of Molecular Biology and Biochemistry, Institute of Biomedical and Health Sciences, Hiroshima University, Hiroshima 734–8553, Japan

## Abstract

OASIS/CREB3L1, an endoplasmic reticulum (ER)-resident transcription factor, plays important roles in osteoblast differentiation. In this study, we identified new crosstalk between OASIS and the hypoxia signaling pathway, which regulates vascularization during bone development. RT-PCR and real-time PCR analyses revealed significant decreases in the expression levels of hypoxia-inducible factor-1α (HIF-1α) target genes such as vascular endothelial growth factor A (VEGFA) in OASIS-deficient (*Oasis*^−/−^) mouse embryonic fibroblasts. In coimmunoprecipitation experiments, the N-terminal fragment of OASIS (OASIS-N; activated form of OASIS) bound to HIF-1α through the bZIP domain. Luciferase assays showed that OASIS-N promoted the transcription activities of a reporter gene via a hypoxia-response element (HRE). Furthermore, the expression levels of an angiogenic factor *Vegfa* was decreased in *Oasis*^−/−^ osteoblasts. Immunostaining and metatarsal angiogenesis assay showed retarded vascularization in bone tissue of *Oasis*^−/−^ mice. These results suggest that OASIS affects the expression of HIF-1α target genes through the protein interaction with HIF-1α, and that OASIS-HIF-1α complexes may play essential roles in angiogenesis during bone development.

The endoplasmic reticulum (ER) is a cellular organelle that plays important roles in protein synthesis, protein folding, and post-translational modifications of secreted proteins^1^,^2^. Environmental and genetic factors, such as ischemia, glucose deprivation, or mutant protein expression, cause the accumulation of misfolded and/or unfolded proteins in the ER lumen. This condition is called ER stress^3^,^4^,^5^. Cells cope with ER stress by activating diverse signals from the ER to the cytoplasm or nucleus, which is termed the unfolded protein response (UPR)^6^,^7^,^8^. The signaling cascades during the UPR are mainly mediated by three major ER stress transducers, PKR-like endoplasmic reticulum kinase (PERK)^9^, inositol-requiring enzyme 1 (IRE1)^10^, and activating transcription factor 6 (ATF6)^11^,^12^. These transducers sense the accumulation of unfolded proteins in the ER lumen and transmit signals for attenuation of ER stress.

Recently, novel types of ER stress transducers have been identified, which are referred to as the OASIS family^13^,^14^,^15^. These proteins are structurally similar to ATF6, possess a basic leucine zipper (bZIP) domain and a transmembrane domain. The bZIP domain contains a basic motif region that mediates sequence specific DNA binding properties, and the leucine zipper that is required to dimerize two DNA binding regions. The OASIS family consists of five members, namely old astrocyte specifically induced substance (OASIS)/CREB3L1^16^, BBF2 human homolog on chromosome 7 (BBF2H7)/CREB3L2^17^, Luman/LZIP/cyclic AMP-response element-binding protein 3 (CREB3)^18^, cyclic AMP-response element-binding protein H (CREBH)/CREB3L3^19^, and androgen-induced bZIP (AIbZIP)/CREB4/Tisp40/CREB3L4^20^. All five members are basically localized to the ER membrane, become transported to the Golgi apparatus in response to various stimuli such as ER stress, and are then processed by Site-1 and Site-2 proteases at their transmembrane domains^21^. The cleaved N-terminal fragments of the OASIS family members move into the nucleus, and act as transcription factors. The unique feature of the OASIS family members is their cell type- or tissue-specific expression patterns. For example, OASIS is preferentially expressed in osteoblasts and astrocytes^13^,^21^, BBF2H7 in chondrocytes^22^, CREBH in liver cells^23^,^24^, and AIbZIP in the testis and prostate^25^,^26^. These observations suggest that the functions of the individual members may be involved in regulation of the UPR in specific organs and tissues.

OASIS is constitutively degraded under normal conditions, but ER stress leads to inhibition of ubiquitination and proteasome-mediated degradation of OASIS by HRD1, which is an ER-resident E3 ubiquitin ligase. Both full-length and cleaved OASIS are significantly stabilized by ER stress, with cleaved OASIS enhancing transcriptional activation of its target genes^27^. Moreover, we previously showed that OASIS is highly expressed in osteoblasts and regulates the transcription of type 1 collagen^21^. Furthermore, we demonstrated that deficiency of OASIS is associated with an osteopenic phenotype^21^, indicating that OASIS plays an essential role in osteogenesis. Recently, OASIS was identified as one of the causative genes for osteogenesis imperfecta in humans^28^. In addition, OASIS is involved in the differentiation of various cells such as goblet cells and astrocytes as well as those in bone tissues^15^,^29^,^30^,^31^. These findings demonstrate that OASIS has pivotal roles in the differentiation of various tissues.

Hypoxia is a condition in which organs or cells are deprived of an adequate oxygen supply. When cells are exposed to hypoxic conditions, adaptive signal transduction pathways are activated, resulting in the secretion of various factors that contribute to angiogenesis^32^,^33^. Recent evidence from basic and clinical research studies has indicated that hypoxia is a strong activator of the UPR^34^,^35^, and that the UPR regulates the expressions of genes that are known to increase hypoxia tolerance and survival in cells^36^,^37^. However, the molecular mechanisms and physiological functions of the UPR during hypoxia are still poorly understood.

Hypoxia-inducible factors (HIFs) are important regulators of cellular homeostatic responses to hypoxia^38^. HIFs contain a Per-ARNT-Sim (PAS) domain essential for interactions with their cofactor proteins, and a basic helix-loop-helix (bHLH) domain required for DNA binding to the hypoxia-response element (HRE) in the promoter region of their target genes^39^,^40^,^41^,^42^,^43^. HIF-1α, one of the HIF subtype proteins, plays key roles in embryonic vascularization, tumor angiogenesis, and pathophysiological ischemic diseases^44^,^45^. The stability of HIF-1α is regulated by hydroxylation of proline residues in its polypeptide sequence. In normoxia, hydroxylation at proline 402 and proline 564 in the HIF-1α protein creates a binding site for von Hippel-Lindau (VHL) protein, an E3 ubiquitin protein ligase^46^,^47^. VHL promotes the ubiquitination of HIF-1α and leads to proteasomal degradation of a HIF-1α subunit^48^. Under hypoxia, the proline hydroxylation in HIF-1α is impaired and ubiquitination of HIF-1α is attenuated, leading to stabilization of HIF-1α. HIF-1α dimerizes with HIF-1β through its bHLH-PAS domain^49^, and binds to the promoter region of target genes such as vascular endothelial growth factor A (VEGFA) to facilitate their expression^40^.

VEGFA is expressed in chondrocytes and osteoblasts as an important regulator of angiogenesis during endochondral ossification^43^,^50^,^51^,^52^. Functional deprivation of VEGFA in developing cartilage causes inhibition of blood vessel invasion into the hypertrophic zone, resulting in impairment of trabecular bone formation and expansion of the hypertrophic zone^53^. Recently, it was reported that ER stress potentiated HIF-1α activity to promote transcription of VEGFA^35^.

In the present study, we found that OASIS is upregulated in a time-dependent manner during hypoxia, and that OASIS binds to HIF-1α through its bZIP domain and promotes the transcription of hypoxia-inducible genes including VEGFA under hypoxic conditions. Furthermore, OASIS deficiency causes impairment of vessel formation in bone tissue.

## Results

### OASIS regulates the expression of HIF-1α target genes

**Figure 1:**
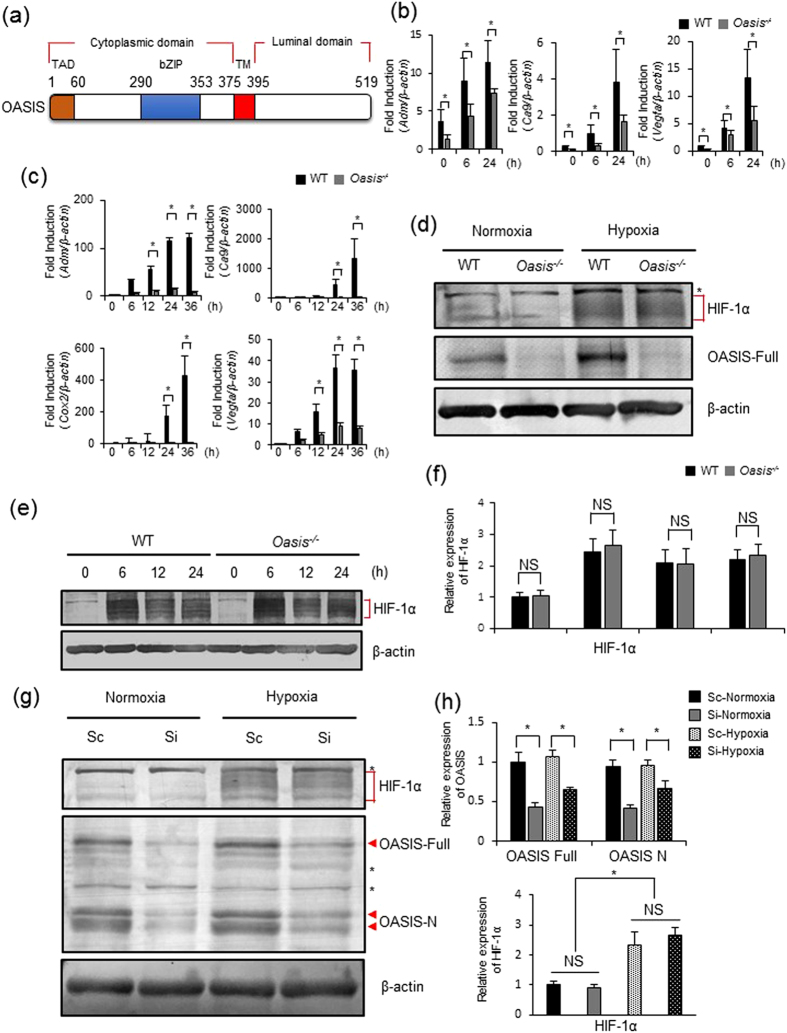
OASIS regulates the expression of HIF-1α target genes. (**a**) Schematic representation of human OASIS protein. (**b**) Real-time PCR analyses of WT and *Oasis*^−/−^ Primary osteoblasts. Cells were exposed to hypoxia (0.2% oxygen) for the indicated time periods (mean ± s.d., *n* = 3; *P < 0.05, *t-*test). Note that the expression levels of the HIF-1α target genes are impaired in *Oasis*^−/−^ cells. (**c**) Real-time PCR analyses of WT and *Oasis*^−/−^ mouse embryonic fibroblasts (MEFs). Cells were exposed to hypoxia (0.2% oxygen) for the indicated time periods (mean ± s.d., *n* = 3; *P < 0.05, *t-*test). (**d**) Western blot analyses for OASIS and HIF-1α. Primary osteoblasts cells were exposed to hypoxia for 6 h. Cell lysates were subjected to western blotting analyses using anti-OASIS and anti-HIF-1α antibodies. Asterisk, nonspecific bands. (**e,f**) Western blot analyses for HIF-1α. WT and *Oasis*^−/−^ MEFs were exposed to hypoxia (0.2% oxygen) for the indicated time periods. A quantitative chart is shown in panel f (mean ± s.d., *n* = 3; NS, not significant *P* > 0.05, *t-*test). (**g,h**) Western blot analyses for OASIS and HIF-1α. MC3T3-E1 cells were transfected with scramble siRNA (Sc) or OASIS-specific siRNA (Si) and exposed to hypoxia for 6 h. Cell lysates were subjected to western blot analyses using anti-OASIS and anti-HIF-1α antibodies. Asterisk, nonspecific bands; arrow, specific bands for OASIS. A quantitative chart is shown in panel h (mean ± s.d., *n* = 3; *P < 0.05, NS, not significant = *P* > 0.05, *t*-test). Full-length blots are presented in the supplementary information (supplementary Figure S5).

OASIS, a type 2 transmembrane bZIP transcription factor (Fig. 1a), plays important roles in the maturation of osteoblasts and bone formation^21^. However, the detailed mechanisms underlying the regulation of osteogenesis by OASIS are not fully understood. Therefore, we carried out a profiling analysis of gene expressions in osteoblasts derived from wild-type (WT) and OASIS-deficient (*Oasis*^−/−^) mice^21^ (database: http://www.ncbi.nlm.nih.gov/geo/query/acc.cgi?acc=GSE18062). Intriguingly, we found that the expressions of the vast majority of HIF-1α target genes were reduced in *Oasis*^−/−^ cells (Supplementary Table 2). To confirm the results of the gene expression profiling, we performed RT-PCR and real-time PCR analyses using WT and *Oasis*^−/−^ mouse embryonic fibroblasts (MEFs) and primary osteoblasts under hypoxic conditions (0.2% oxygen). The expression levels of HIF-1α target genes, such as *Adm, Ca9* and *Vegfa* were significantly reduced in *Oasis*^−/−^ cells compared with WT cells (Fig. 1b,c, Supplementary Figure S1a,b). Next, we examined whether OASIS affects the expression level of HIF-1α. Western blot analyses of cell lysates from WT and *Oasis*^−/−^ MEFs cultured under normoxia or hypoxia showed no significant difference in the protein levels of HIF-1α between WT and *Oasis*^−/−^ MEFs (Fig. 1e,f). Moreover, we checked the expression levels of HIF-1α in the primary osteoblasts and osteoblastic cell line MC3T3-E1 in which OASIS was knocked down by siRNA transfection. Similar to the case with MEFs, no difference in the HIF-1α protein levels was observed between OASIS-knockdown MC3T3-E1 cells and control cells or *Oasis*^−/−^ primary osteoblast and WT osteoblasts (Fig. 1d,g,h). These data suggest that OASIS deficiency causes downregulation of HIF1α target gene expressions without any impact on HIF-1α expression.

### OASIS is activated by hypoxia and interacts with HIF1α

**Figure 2:**
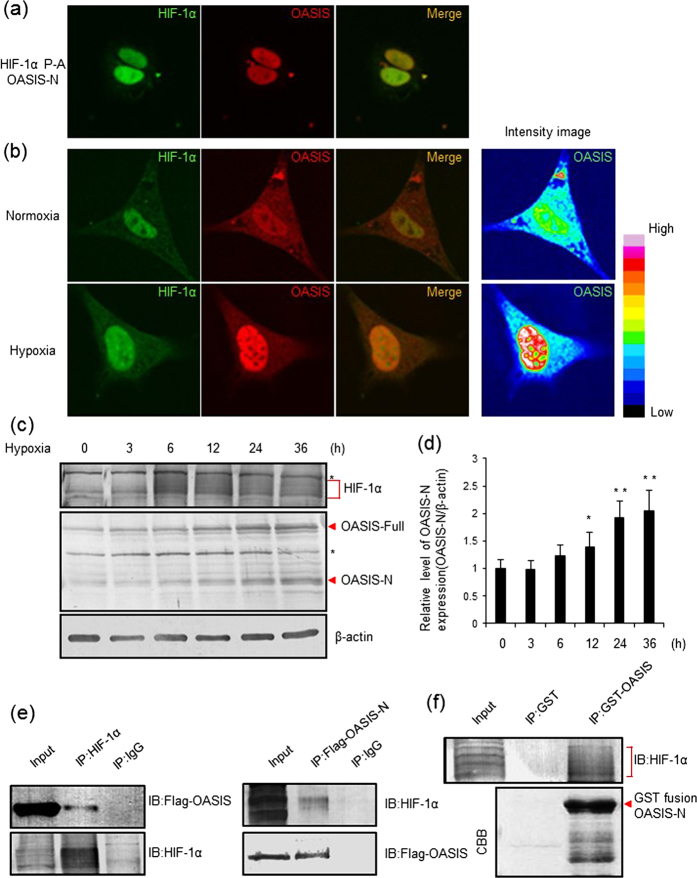
OASIS is activated by hypoxia and interacts with HIF-1α. (**a**) Immunofluorescence staining for HIF-1α and OASIS. HEK293T cells were cotransfected with HIF-1α P402A/P564A (HIF-1α P-A; constitutively activated stabilized form) and OASIS N-terminus (OASIS-N) expression plasmids. Both HIF-1α P-A (green) and OASIS-N (red) are detected in the nucleus. (**b**) Immunofluorescence analyses of endogenous HIF-1α (green) and OASIS (red) in MC3T3-E1 cells under normoxia or hypoxia (0.2% oxygen, 24 h). Intensity images showed increasing nuclear intensity of OASIS in the MC3T3-E1 under hypoxia. (**c,d**) Western blot analyses for HIF-1α and OASIS. MC3T3-E1 cells were exposed to hypoxia (0.2% oxygen) for the indicated time periods. Asterisk, nonspecific bands; arrow, specific bands for OASIS. A quantitative chart is shown in panel d (mean ± s.d., *n* = 3; *P < 0.05; *t-*test). (**e**) Co-IP followed by wester*n* blot analyses of HIF-1α and OASIS. HEK293T cells were transfected with Flag-tagged OASIS-N and exposed to hypoxia. Cell lysates were subjected to Co-IP assays with anti-Flag or anti-HIF-1α antibodies. The IP samples were subjected to western blotting with anti-HIF-1α and anti-OASIS antibodies. (**f**) GST pulldown assays to examine the protein interaction of OASIS and HIF-1α. Recombinant GST-OASIS-N or GST proteins were produced in *E. coli* BL21 cells. Recombinant proteins were incubated with cell lysates from hypoxia-exposed MC3T3-E1 cells. Pulldown was carried out using glutathione-Sepharose beads and western blotting with anti-HIF-1α antibodies was performed. Coomassie Brilliant Blue (CBB) staining confirmed the existence of GST-OASIS-N in pulldown samples. Full-length blots are presented in the supplementary information (supplementary Figure S6).

We investigated the subcellular localizations of OASIS and HIF-1α. HEK293T cells were cotransfected with expression plasmids for the OASIS N-terminus (OASIS-N; active form) and a stabilized form of HIF-1α that has mutations in hydroxylation sites and is constitutively activated (HIF1α P-A; P402A/P564A)^54^,^55^. Immunofluorescence staining with anti-OASIS and anti-HIF-1α antibodies showed that OASIS-N and HIF-1α P-A were both localized to the nucleus (Fig. 2a). To further understand the physiological distribution, we examined the localizations of endogenous OASIS and HIF-1α in MC3T3-E1 cells under normoxia or hypoxia. As previously reported^56^, HIF-1α was detected in the nucleus of MC3T3-E1 cells under both normoxia and hypoxia (Fig. 2b, left panels). On the other hand, although OASIS was diffusively localized in the cytoplasm and nucleus under normoxia, the signals for OASIS were strongly detected in the nucleus under hypoxia, and overlapped with those of HIF-1α (Fig. 2b, middle panels and the intensity images, Supplementary Figure S2a–c). These observations suggest that OASIS might be cleaved and activated in the hypoxic conditions, and may interact with HIF-1α in the nucleus. To confirm that OASIS is activated by hypoxia, we conducted western blotting analyses of OASIS in MC3T3-E1 cells exposed to hypoxia for different time periods. As expected, the cleaved OASIS N-terminal fragment gradually increased within 12–36 h after hypoxic exposure (Fig. 2c,d, Supplementary Figure S2d). Additionally, overexpression of OASIS in HEK293T showed that the increased amount of OASIS leads to an increase in the relative expression of nuclear HIF-1α (Supplementary Figure S3a,b). Note that the overexpression of OASIS-N might influence the localization and the consumption rate of HIF-1α.

Next, we performed coimmunoprecipitation (Co-IP) experiments to examine the physical interaction between OASIS-N and HIF-1α. We found that HIF-1α was coprecipitated with OASIS-N, and conversely, that OASIS-N was coprecipitated with HIF-1α (Fig. 2e, Supplementary Figure S2e). These results indicate that OASIS-N and HIF-1α physically interact with each other. To further verify the direct interaction between HIF-1α and OASIS-N, a glutathione S-transferase (GST) pulldown assay was performed. The *in vitro* GST pulldown assay confirmed a direct interaction between GST-OASIS-N and HIF-1α (Fig. 2f). These data indicate that OASIS becomes activated under hypoxia and has the potential to bind to HIF-1α and affect the transcription of HIF-1α target genes.

### OASIS promotes the transcription of HIF-1α target genes through an HRE

**Figure 3:**
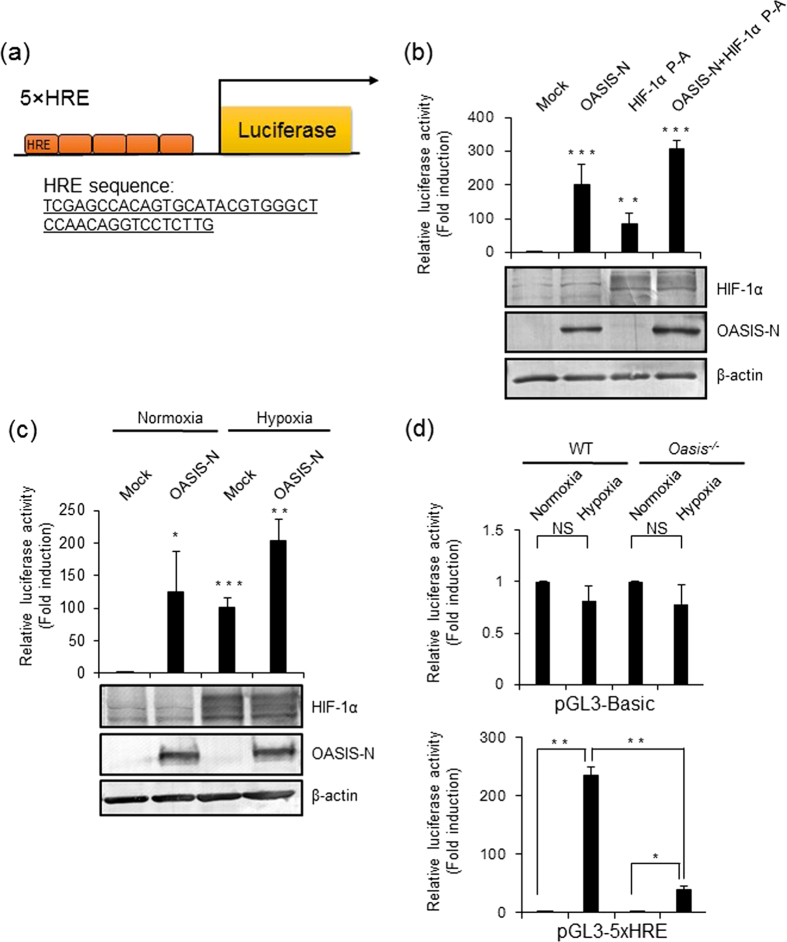
OASIS enhances the promoter activity of the HRE. (**a**) Schematic representation of the 5 × HRE reporter construct and the inserted sequence of the HRE. (**b**) Luciferase reporter analyses of the 5 × HRE promoter. HEK293T cells were cotransfected with the pGL3-5 × HRE reporter plasmid and the OASIS-N and/or HIF-1α expression plasmids. At 24 h after transfection, the luciferase activities were measured (mean ± s.d., *n* = 6; **P < 0.01, ***P < 0.001, *t-*test). Cell lysates were subjected to western blotting with anti-HIF-1α or anti-OASIS antibodies to check the protein levels of overexpressed HIF-1α or OASIS. (**c**) Luciferase reporter analyses for the 5 × HRE promoter in response hypoxia. HEK293T cells were transfected with the pGL3-5 × HRE reporter plasmid and the OASIS-N or Mock plasmids. At 12 h after transfection, cells were exposed to the normoxia or hypoxia for 12 h (mean ± s.d., *n* = 3; *P < 0.05, **P < 0.01, ***P < 0.001, *t-*test). (**d**) Luciferase reporter analyses of the 5 × HRE promoter under hypoxia. WT or *Oasis*^−/−^ MEFs were transfected with pGL3-Basic or pGL3-5 × HRE reporter plasmids and exposed to normoxia or hypoxia for 24 h (mean ± s.d., *n* = 3; *P < 0.05, **P < 0.01, *t-*test). Note that the reporter activities of *Oasis*^−/−^ cells under hypoxia are significantly suppressed compared with those in WT cells. Full-length blots are presented in the supplementary information (supplementary Figure S7).

It is known that HIF-1α promotes the transcription of its target genes through an HRE^56^. To assess whether OASIS-N affects the transcription of HIF-1α target genes through an HRE, we generated a pGL3-5 × HRE reporter plasmid (HRE sequence: 5′-TCGAGCCACAGTGCATACGTGGGCTCCAACAGGTCCTCTTG-3′) (Fig. 3a) as previously reported^57^, and performed luciferase assays. Compared with mock-transfected cells, the reporter activities of cells transfected with OASIS-N or HIF-1α were significantly increased (Fig. 3b). When cells were cotransfected with both OASIS-N and HIF-1α, the reporter activities were enhanced in an additive manner (Fig. 3b). Furthermore, we examined the reporter activities in response to hypoxia or cobalt dichloride (CoCl_2_), which mimics the hypoxic conditions and induces stabilization of HIF-1α. In a similar manner to the above-described findings, hypoxia and CoCl_2_ increased the reporter activities in control cells (Fig. 3c, Supplementary Figure S2f). Furthermore, expression of OASIS-N in hypoxia or CoCl_2_ treated cells reinforced the reporter activities in an additive manner (Fig. 3c, Supplementary Figure S2f). To investigate the effect of endogenous OASIS on the HRE, we performed luciferase assays using WT and *Oasis*^−/−^ MEFs. Upon introduction of the pGL3-Basic control reporter plasmid lacking a promoter region, the reporter activities in WT and *Oasis*^−/−^ MEFs showed no difference, even under hypoxia (Fig. 3d, top). In contrast, the reporter activities in WT MEFs transfected with the pGL3-5 × HRE reporter plasmid were dramatically increased in response to hypoxia (Fig. 3d, bottom). However, the luciferase activities under hypoxia were remarkably suppressed in *Oasis*^−/−^ MEFs (Fig. 3d, bottom). Thus, OASIS is likely to regulate the expression of HIF-1α target genes through the HRE in a coordinated manner with HIF-1α.

### The bZIP domain of OASIS binds to the bHLH domain of HIFs

**Figure 4:**
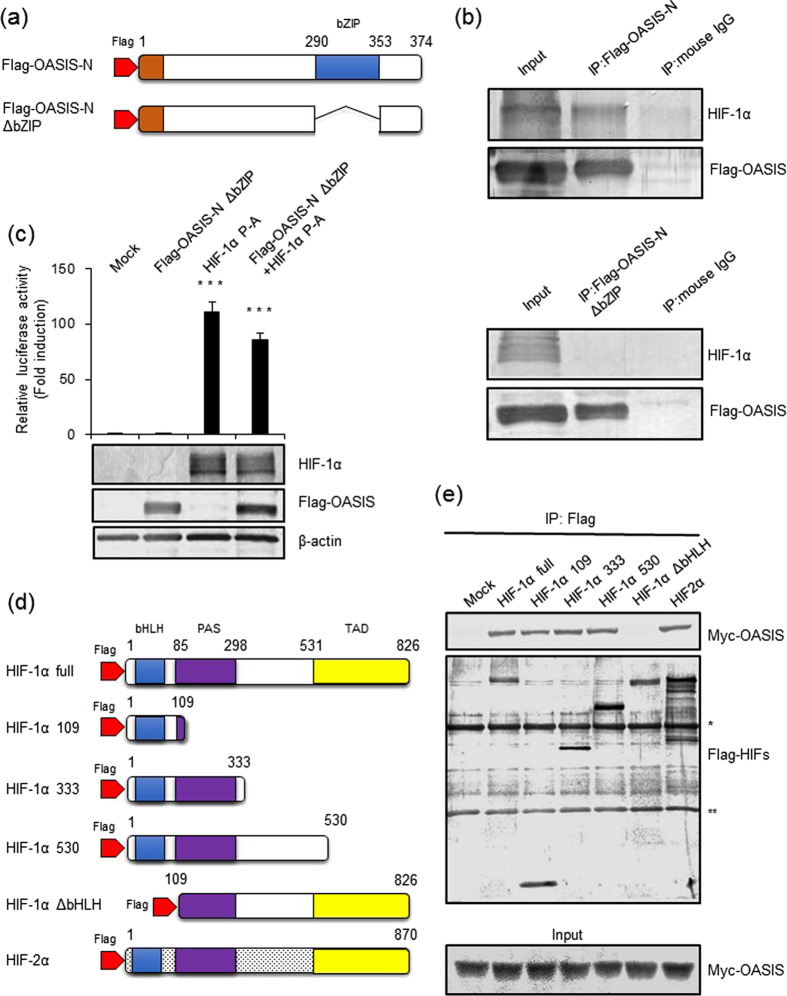
bZIP domain of OASIS binds to the bHLH domain of HIFs. (**a**) Schematic representation of the Flag-OASIS-N and Flag-OASIS-N ΔbZIP constructs. (**b**) Co-IP and western blot analyses. HEK293T cells were tr**a**nsfected with HIF-1α P-A and FLAG-OASIS-N or FLAG-OASIS-N ΔbZIP expression plasmids. Cell lysates were subjected to Co-IP with anti-Flag antibodies, and the IP samples were subjected to western blotting with anti-HIF-1α and anti-OASIS antibodies. (**c**) Luciferase reporter analyses of the 5 × HRE promoter. HEK293T cells were cotransfected with a pGL3-5 × HRE reporter plasmid and FLAG-OASIS-N ΔbZIP and/or HIF-1α expression plasmids (means ± s.d., *n* = 6; ***P < 0.001, *t-*test). (**d**) Schematic representations of the Flag-tagged HIF-1α deletion mutant and Flag-tagged HIF-2α expression plasmids. (**e**) Co-I*P* and western blot analyses using HIF-1α mutants and HIF-2α. HEK293T cells were transfected with the Myc-tagged OASIS-N expression plasmid together with a series of HIF-1α mutant or HIF-2α expression plasmids. Co-IP of cell lysates was performed with anti-Flag antibodies, and the IP samples were subjected to western blotting analyses with anti-Myc and anti-Flag antibodies (*IgG-H fragments, **IgG-L fragments). Full-length blots are presented in the supplementary information (supplementary Figure S8).

To examine whether the bZIP region of OASIS is important for the interaction between OASIS and HIF-1α, we constructed an expression plasmid for bZIP domain-deleted OASIS with a Flag-tag at the N-terminus (Flag-OASIS-N ΔbZIP) (Fig. 4a), and performed Co-IP experiments. Co-IP and western blot analyses showed that HIF-1α was coprecipitated with Flag-OASIS-N (Fig. 4b, top), but not with Flag-OASIS-N ΔbZIP (Fig. 4b, bottom). Moreover, luciferase assays with the Flag-OASIS-N ΔbZIP expression plasmid and pGL3-5 × HRE reporter plasmid revealed that Flag-OASIS-N ΔbZIP abolished induction of the promoter activity through the HRE (Fig. 4c). When the Flag-OASIS-N ΔbZIP and HIF-1α expression plasmids were cotransfected, the reporter activities were not enhanced in an additive manner compared with introduction of HIF-1α only (Fig. 4c). To further examine the binding region of OASIS in HIF-1α, we generated four kinds of Flag-tagged HIF1α deletion constructs; HIF-1α ΔbHLH (109-826aa), HIF-1α 109 (1-109aa), HIF-1α 333 (1-333aa) and HIF-1α 530 (1-530aa) and a construct of HIF-2α, which is an important HIF-1α homologous protein engaged in the hypoxia pathway (Fig. 4d). Co-IP experiments using a Myc-tagged OASIS-N expression plasmid and the HIF-1α mutant series or HIF-2α construct showed that only HIF-1α ΔbHLH was not coprecipitated with OASIS-N, while the remaining HIF constructs were all coprecipitated with OASIS-N (Fig. 4e). These results indicate that the OASIS bZIP domain interacts with the bHLH domain of HIF-1α and HIF-2α.

### OASIS plays a crucial role in vascularization during bone development

**Figure 5:**
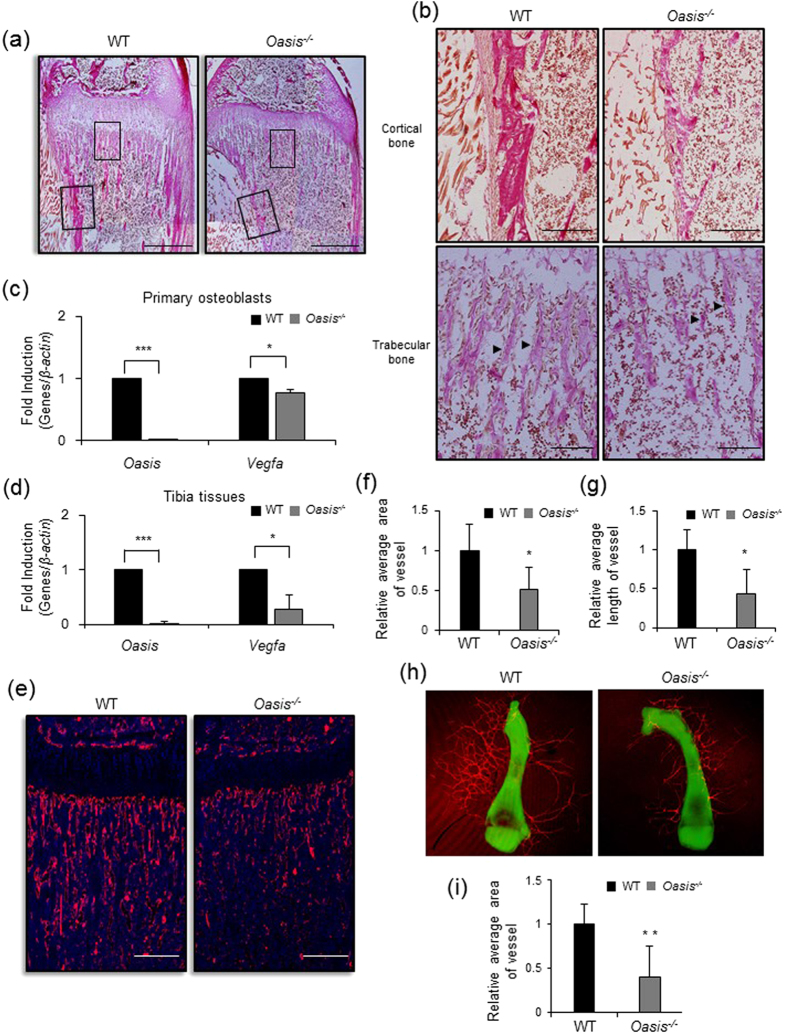
OASIS plays a crucial role in vascularization during bone development. (**a**) Van Gieson staining of undecalcified frozen tibial sections from WT and *Oasis*^−/−^ mice (28 days-old). Van Gieson staining only detects the collagen matrix in bone tissues. Note the dramatic decrease in the collagen fibril content in *Oasis*^−/−^ mice compared with that in WT mice. Scale bar, 500 μm. (**b**) Higher magnification images of cortical bone and trabecular bones in the boxes shown in panel a. Arrowheads indicate trabecular bones. Scale bar, 200 μm. (**c,d**) Real-time PCR analyses of OASIS and VEGFA expressions in WT and *Oasis*^−/−^ primary cultured osteoblasts (C) and tibia tissues (D). The expression of *Vegfa* mRNA is significantly decreased in *Oasis*^−/−^ cells and bone tissues (mean ± s.d., *n* = 3; *P < 0.05, ***P < 0.001, *t-*test). (**e–g**) Immunofluorescence staining with anti-CD31 antibody in undecalcified frozen tibial sections from WT and *Oasis*^−/−^ juvenile mice (28 days-old). Note that retarded vascularization is observed in the *Oasis*^−/−^ tibia. Quantitation of the vessel areas (**f**) and the vessel lengths (**g**) are indicated in panel f and g (mean ± s.d., *n* = 5; *P < 0.05, *t-*test). Scale bar, 200 μm. (**h,i**) Absence of OASIS decreases angiogenesis in metatarsal explants. Metatarsal explants stained for endothelial cells (red, CD31); P0.5 mouse metatarsals derived from WT and *Oasis*^−/−^ mice were cultured for 15 days before fixation. Green color showed the pattern of mosue metatarsal. Quantitation of the vessel areas are indicated in panel h (mean ± s.d., *n* = 6; **P < 0.01, *t-*test).

Previously, we observed systemic osteopenia in *Oasis*^−/−^ mice that was not limited to the femurs or tibias^21^. Reductions in bone mass in the skeletal system cause marked fragility of bone, and bone fractures are often observed in the vertebrae and long bones of *Oasis*^−/−^ mice. Van Gieson staining of undecalcified frozen tibial sections from juvenile mice revealed the osteopenic phenotype of *Oasis*^−/−^ mice (Fig. 5a). The collagen density in cortical bone was decreased in *Oasis*^−/−^ mice (Fig. 5b, top), and the thickness of trabecular bone was markedly reduced (Fig. 5b, bottom).

A recent study showed that deprivation of VEGFA inhibits blood vessel formation in the marrow cavity^53^, resulting in reduced trabecular bone and reflecting a similar phenotype to that of *Oasis*^−/−^ mice. VEGFA is an important target of HIF1α involved in angiogenesis, and our present findings indicating that OASIS has an irreplaceable function in the HIF-1α pathway suggest that OASIS regulates bone angiogenesis by mediating the HIF-1α-VEGFA signaling cascade. To gain further insight into the defective angiogenesis in *Oasis*^−/−^ mice, we examined the VEGFA gene expression in primary cultured osteoblasts (Fig. 5c) and bone tissues (Fig. 5d). Real-time PCR analyses showed that the expression levels of VEGFA were substantially suppressed in *Oasis*^−/−^ osteoblasts and *Oasis*^−/−^ bone tissues (Fig. 5c,d).

To examine the organization of blood vessels in bone, we carried out immunofluorescence staining using an antibody against CD31, also known as platelet endothelial cell adhesion molecule (PECAM), a specific marker for endothelial cells^58^, on undecalcified frozen tibial sections. In *Oasis*^−/−^ mice, the CD31-positive vascular network development was significantly reduced compared with that in WT mice (Fig. 5e, Supplementary Figure S4a-f). The vessel quantity were apparently decreased in *Oasis*^−/−^ mice compared with WT mice (Fig. 5f,g). To determine the effect of OASIS on bone angiogenesis in a more physiological model, we performed an *ex vivo* angiogenesis assay using P0.5 mouse metatarsals from WT and *Oasis*^−/−^ mice. This assay showed that angiogenesis in fetal mouse metatarsals was remarkably decreased in *Oasis*^−/−^ mice compared with WT mice (Fig. 5h,i).

## Discussion

**Figure 6:**
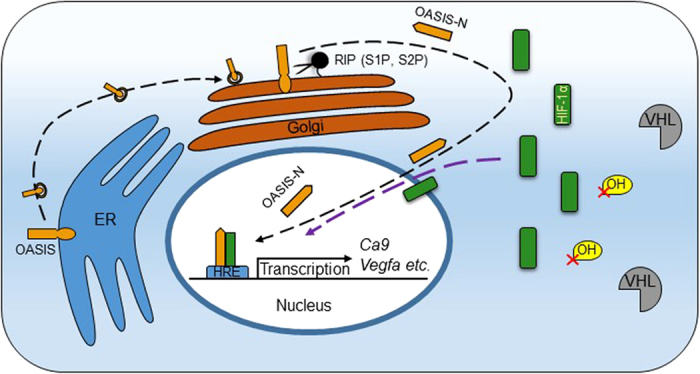
Model for the mechanisms of bone vascularization by OASIS in osteoblasts. Under hypoxic conditions, OASIS is transported from ER to the Golgi apparatus, and undergoes processing by S1P and S2P proteases. The cleaved OASIS N-terminus and stabilized HIF-1α form a heterodimer in nucleus and synergistically promote the transcriptional activation of HIF-1α target genes through the hypoxia-response element. VHL: von Hippel-Lindau; RIP: regulated intramembrane proteolysis; ER: endoplasmic reticulum; OH: hydroxylation; HRE: hypoxia-response element. (Figure was drawn by Min Cui).

The present study has significantly extended our understanding of the physiological roles of OASIS, an ER stress transducer, in the hypoxia stress response. The following evidence demonstrates that OASIS regulates the expression of HIF-1α target genes by interacting with HIF-1α under hypoxia: 1) the expression of HIF-1α target genes was significantly suppressed in *Oasis*^−/−^ cells (Fig. 1b,c); 2) OASIS was activated in response to hypoxia (Fig. 2c,d); 3) OASIS interacted with HIF-1α and HIF-2α directly (Figs 2e,f and 4e); 4) OASIS N-terminus might be involved in the nuclear localization of HIF-1α (Supplementary Figure 3a,b); 5) OASIS strongly promoted gene transcription activities through the HRE (Fig. 3b); and 6) the absence of OASIS impaired the vascular network development mediated by the HIF-1α-VEGF signaling cascade in bone tissues (Fig. 5e,h). We suppose the following mechanism for the activation and physiological roles of OASIS under hypoxia. OASIS is transported from the ER to the Golgi apparatus, and undergoes processing by S1P and S2P proteases. The activated OASIS N-terminus then interacts with HIF1α to directly promote the expression of target genes such as *Vegfa* through the HRE (Fig. 6). It is known that OASIS is activated by ER stress^59^. On the other hand, several lines of evidence have shown that hypoxic conditions can induce ER stress in several cell types^60^. Therefore, the mechanism for OASIS activation during hypoxia might be caused by hypoxia-induced ER stress. Taken together, the present findings show that OASIS plays essential roles in bone development, not only through the production of bone matrix by regulating Col1 expression directly^21^, but also through the promotion of bone angiogenesis by interacting with the transcription factor HIF-1α.

We previously reported that the bZIP transcription factor OASIS regulates the expression of VEGFA through binding to a CRE-like core sequence (ACGT) in the VEGFA promoter region^61^. It is also known that HIF-1α regulates VEGFA expression through the HRE^62^. The HRE sequence (G/ACGTG) is similar to the binding sequence of OASIS (i.e. ACGT). Indeed, we demonstrated that the activated OASIS N-terminus interacts with HIF-1α and enhances the promoter activity through the HRE sequence (Figs 2e,f and 3b,c). A recent study showed that one of the bZIP family proteins, XBP1, can interact with HIF-1α in particular cell types^54^. It is known that the bZIP domains in the family members resemble one another structurally, and could potentially bind to each other^63^. These findings raise the further possibility of interactions between bZIP family proteins and bHLH family proteins to synergistically control their target gene expressions in several pathophysiological conditions. HIF-1α requires heterodimerization with aryl hydrocarbon receptor nuclear translocator (Arnt/HIF-1β) in the nucleus to promote its target gene transcriptions^64^. Although we demonstrated direct interactions of OASIS with HIF-1α and HIF-2α, the interaction between OASIS and HIF-1β is still unknown, and the relevance of interactions between HIFs and other kinds of OASIS family proteins has not yet been studied. Further investigations are required to elucidate the functions and mechanisms of the interactions between bZIP proteins and HIFs under physiological and pathological conditions in cells.

A previous report suggested that HIF-1α promotes angiogenesis and osteogenesis by elevating VEGF levels in osteoblasts in bone development^65^. During bone development, osteoblasts are exposed to mild hypoxic conditions, followed by attenuated hydroxylation of HIF-1α by VHL, resulting in elevated VEGFA expression^66^. VEGFA induces migration of endothelial cells to the oxygen-deficient zone to provide oxygen and nutrients for osteoblasts and chondrocytes and help their maturation and proliferation^50^,^51^. A novel finding regarding OASIS function in the present study was the involvement of OASIS in angiogenesis in bone tissue mediated by the HIF-1α pathway. OASIS deficiency led to imperfect vascularization during bone development. Poorly produced blood vessels cannot provide sufficient nutrition to osteoblasts to develop trabecular bones, resulting in the formation of a vulnerable skeletal system. OASIS also regulates the expression of type I collagen, one of the bone matrix proteins^21^. Therefore, OASIS might engage in different phenomena during bone development by interacting with different binding partners.

In summary, we have shown that OASIS modulates the HIF-1α signaling pathway to contribute to bone vascularization. Our findings indicate that OASIS-HIF-1α complexes might improve angiogenesis during bone formation, wound healing, and granulated tissue formation. Furthermore, OASIS-HIF complexes may provide better solutions in therapies for angiodysplasia in osteogenesis imperfecta.

## Materials and Methods

### Cell culture and reagents

MEF and HEK293T cells were grown in Dulbecco’s modified Eagle’s medium (Gibco, USA) containing 10% fetal calf serum (Sigma, USA). MC3T3-E1 (mouse osteoblast) cells were maintained in α-modified eagle medium (Gibco) containing 10% FCS. We used 1 μM MG132 (WAKO, Japan), a proteasome inhibitor, to inhibit protein degradation, and used 150 mM cobalt dichloride (WAKO) to mimic hypoxia.

### RT-PCR and Real-time PCR

Total RNA was extracted from cells using ISOGEN (WAKO) according to the manufacturer’s protocol. First-strand cDNA was synthesized in a 20 μl of reaction volume using a random primer (TAKARA, Japan) and 1 μl reverse transcription enzyme M-MLV-RT (Invitrogen, USA). PCR was performed using each specific primer set in a total volume of 20 μl containing 10 pmol of each primer, 4 μmol dNTPs, 1 unit of Paq5000 DNA polymerase (Agilent, USA), and 1 × PCR buffer. Primer sequences are summarized in supplemental Table S1. The PCR products were resolved by electrophoresis on a 4.8% acrylamide gel. For the real-time PCR analyses, SYBR FAST qPCR Master Mix (Kapa Biosystems, USA) was used, and PCR products were measured by LightCycler 480 II (Roche, Swiss).

### Antibodies, Western blotting

Mouse monoclonal anti-OASIS antibodies were generated as described previously^21^. For western blotting and immunofluorescence, the following antibodies were used: anti-β-actin (Sigma; 1:3000), anti- HIF-1α (Novus, USA; 1:3000), anti-Flag M2 (Sigma; 1:2000), anti-Myc (MBL, USA; 1:3000), anti-CD31 (BD, USA; 1:1000). For western blot analyses, proteins were extracted from cells using cell extraction buffer containing 20 mM Tris-HCl (pH 7.5), 500 mM NaCl, 10 mM MgCl_2_, 2 mM EDTA, 10% glycerol, 1% Triton X-100, 2.5 mM β-glycerophosphate, 1 mM NaF, 1 mM DTT, and 1 mM complete protease inhibitors (WAKO) at 4 °C. After centrifugation, soluble protein in the lysates were quantified. Samples were loaded onto 8–12% sodium dodecyl sulfate-polyacrylamide gels. Protein-equivalent samples were subjected to western blotting.

### Cytosol/Nuclear extraction

Cells were harvested by cell-scraper from 6 well plate, and cytosol proteins were extracted using 100 μl of cytosol lysis buffer1 (10 mM Hepes (pH 7.9), 10 mM KCl, 0.1 mM EDTA, 0.2% NP-40, 1 mM DTT, and complete protease inhibitors (WAKO)) at 4 °C for 15 min. Then 6.25 μl of 10% NP-40 was added (vortex for 15 seconds at maximum speed). Cell lysates were centrifuged at 4 °C, 13000 rpm for 10 seconds, then the supernatant were harvested as cytosol fraction. Pellets were washed with 500 μl of cytosol lysis buffer1 for 5 times, then nuclear proteins were extracted using 50 μl nuclear lysis buffer2 (20 mM Hepes (pH 7.9), 420 mM NaCl, 0.1 mM EDTA, 1.5 mM MgCl_2_, 25% Glycerol, 1 mM DTT, and 1 mM complete protease inhibitors (WAKO)) at 4 °C for 30 min. Lysates were centrifuged at 4 °C, 15000 rpm for 15 min, the supernatant was harvested as the nuclear fraction.

### Histological staining and immunofluorescence

Anesthetized mice were perfused with 4% paraformaldehyde in phosphate-buffered saline (PFA/PBS) containing 20% sucrose, and their legs were dissected. The specimens were fixed in 4% PFA/PBS containing 20% sucrose for 3 h, embedded in SCEM (Section-Lab, Japan), and frozen inn-hexane cooled with dry ice. Undecalcified frozen sections at a thickness of 4 μm were obtained according to Kawamoto’s film method using tungsten carbide blades, either TC-65 (Leica Microsystems, Japan) or SL-T35 (Section-Lab), and adhesive films (Section-Lab)^67^. After washing with ethanol and PBS, the sections were fixed in 4% PFA/PBS for 5 min. After blocking with 3.2% skim milk/PBS, the sections were incubated at 4 °C with primary antibodies for overnight (more than 12 h), washed and then incubated with appropriate secondary antibodies conjugated with Alexa Fluor 568 (Life Technologies, USA). Nuclei were counterstained with 4′,6-diamidino-2-phenylindole (DAPI) (Sigma). For immunostaining, MC3T3-E1 or HEK293T cells were seeded at 1 × 10^4^ cells per well on the culture cover glass (MATSUNAMI, Japan), then the cells were transfected and/or treated with reagents for 24 h. Cells were fixed with 4% paraformaldehyde in PBS for 2 h at 4 °C, washed with PBS and incubated with primary antibodies with blocking buffer (3% BSA, 0.3% Triton X-100 in PBS) for overnight (more than 12 h). Cells were washed and then incubated with appropriate secondary antibodies conjugated with Alexa Fluor 568 and/or FITC (Life Technologies) with blocking buffer for 2 h (in dark conditions). Cells were washed with PBS and the cover glass were mounted by fluorescent mounting medium (DAKO, USA).

### Transfection and Co-Immunoprecipitation

1 × 10^6^ cells of HEK293T cells or MC3T3-E1 cells were seeded in 60 mm dish. Cells were transfected with each expression plasmid using ScreenFect A (WAKO) according to the manufacturer’s protocol. After 36 h of transfection, cells were lysed with 300 μl cell extraction lysis buffer (20 mM Tris–HCl (pH 7.4), 10 mM MgCl_2_, 2 mM EDTA, 10% glycerol, 1% Triton X-100, 2.5 mM β-glycerophosphate,1 mM NaF, 1 mM DTT, and protease inhibitor cocktail (WAKO)) containing 500 mM NaCl for 30 minutes on ice (vortex for 10 seconds at maximum speed every 10 minutes), and then 700 μl cell extraction lysis buffer without NaCl was added, followed by cell lysates were incubated for 30 minutes on ice (vortex for 10 seconds at maximum speed every 10 minutes). Cells were centrifuged at 4 °C, 15000 rpm for 20 minutes. Cell lysates were pre-cleared by 20 μl rProtein G Agarose beads (Invitrogen) for 1 h at 4 °C with gentle rotating. Specific antibodies or IgG produced in rabbit or mouse were added together with 20 μl rProtein G Agarose beads, respectively. Cell lysates were incubated at 4 °C for overnight. Then lysates were centrifuged at 500 g to recover the immune complex associated with the beads. The beads complexes were washed five times by gently resuspending in the cell extraction lysis buffer containing 150 mM NaCl, followed by centrifugation at 500 g. At last, 60 μl 2 × laemmli SDS-polyacrylamide gel electrophoresis sample buffer was added and incubated for 10 minutes. Total cell extracts, and immunoprecipitates were subjected to western blotting.

### GST pulldown assay

Recombinant GST or OASIS N-terminus fused to GST (GST-OASIS-N) proteins were prepared from *E. coli* BL21 strain transformed with the expressing vectors for GST only or OASIS N-terminus fused to GST. Recombinant GST/GST-OASIS-N proteins were purified using a glutathione Sepharose 4B beads (GE Healthcare, USA) with standard protocols. One hundred nanograms of GST/GST-OASIS-N proteins were incubated with the one milligram of whole cell lysates from MC3T3-E1 exposed to hypoxia within 1 ml cell extraction lysis buffer at 4 °C for overnight. The precipitated proteins were eluted by adding 2 × laemmli SDS sample buffer and detected by western blotting with anti-HIF-1α antibodies.

### RNA interference

MC3T3-E1 cells were transfected by Lipofectamine RNAiMAX (Invitrogen) according to the manufacturer’s protocols. The transfected cells were incubated at 37 °C for 24 h, then cultured under the hypoxic conditions, and harvested for western blotting. The sequence for siRNA was as follows; (OASIS:5′-GAAAUGAGCCAGUUUCUCAdTdT-3′).

### Luciferase assay

HEK293T cells and MEFs plated onto 24-well plates were transfected with 0.2 μg of a reporter plasmid carrying the firefly luciferase gene and 0.02 μg of the reference plasmid pRL-SV40 carrying the *Renilla* luciferase gene under the control of the SV40 enhancer and promoter with or without 0.2 μg of the OASIS-N and HIF-1α expression plasmids. After 24 h of transfection, cells were lysed in 200 μl of Passive Lysis buffer (Promega, Germany). Firefly luciferase and *Renilla* luciferase activities in 20 μl of cell lysates were measured using the Dual-Luciferase Reporter Assay System (Promega) and a luminometer (Promega). Relative activity was defined as the ratio of firefly luciferase activities to *Renilla* luciferase activities. Twenty microliter of cell lysates were used for western blotting.

### Metatarsal angiogenesis assay

Metatarsals were removed from P0.5 WT and *Oasis*^−/−^ mice, and dissected under a microscope. The isolated metatarsals were cultured in 24-well plates in 150 μl of α-MEM supplemented with 10% (v/v) heat-inactivated fetal bovine serum for 72 h. Cultures were performed in sextuplet and each experiment was repeated at least twice. After 72 h, medium was replaced by 250 μl fresh medium. Metatarsals were cultured for 12 days and medium was replaced every 3 days. At the end of the culture period, metatarsals were fixed in zinc macrodex formalin (ZnMF: 0.1 mM Tris-acetate, pH 4.5, 0.5% ZnCl_2_, 0.5% Zn acetate, 5% dextran, and 3.8% paraformaldehyde) fixative for 20 minutes at room temperature and subsequently stained for anti-CD31 as the immunostaining protocol.

### Quantification of western blot and immunostaining

The quantification of western blotting was performed by Quantity one software. The quantification of immunostaining to measure the vessel’s area were performed by photoshop (Adobe) software using color threshold and measured the number of pixels as the vessel area.

### Microarray analysis

Experimental sample RNAs (calvaria of wild-type and *Oasis*^*−/−*^ mice at postnatal day 4) were isolated using RNeasy(Qiagen) and analysed using Mouse 385 K Array (MM8 60mer expr) and MouseGenome 430 2.0 Array (Affymetrix) by NimbleGen Systems, Inc. and MiyazakiPrefectural Industrial Support Foundation, respectively.

### Statistical analyses

Data are indicated as mean ± s.d. from at least three independent experiments for each experimental condition. Student’s *t*-test was performed to calculated P values. *P* < 0.05 was defined as significant.

## Additional Information

**How to cite this article**: Cui, M. *et al.* OASIS modulates hypoxia pathway activity to regulate bone angiogenesis. *Sci. Rep.* **5**, 16455; doi: 10.1038/srep16455 (2015).

## Supplementary Material

Supplementary Information
