# Supplementary material for: OASIS modulates hypoxia pathway activity to regulate bone angiogenesis
Source: Sci Rep. 2015 Nov 12;5:16455. doi: 10.1038/srep16455 (PMC4642342; doi:10.1038/srep16455)
Supplement: Supplementary Information [file srep16455-s1.pdf]

Supplementary information for

OASIS modulates hypoxia pathway activity to regulate bone angiogenesis

Min Cui[1], Soshi Kanemoto[1], Xiang Cui[1], Masayuki Kaneko[1], Rie Asada[1], Koji Matsuhisa[1], Keiji Tanimoto[2], Yuki Yoshimoto[3], Chisa Shukunami[3] & Kazunori Imaizumi[1]*

[1]Department of Biochemistry, Institute of Biomedical and Health Sciences, Hiroshima University, Hiroshima 734-8553, Japan

[2]Department of Radiation Medicine, Research Institute for Radiation Biology and Medicine, Hiroshima University, Hiroshima 734-8553, Japan

[3]Department of Molecular Biology and Biochemistry, Institute of Biomedical and Health Sciences, Hiroshima University, Hiroshima 734-8553, Japan

*Corresponding author. E-mail: imaizumi@hiroshima-u.ac.jp

Supplementary Figure S1

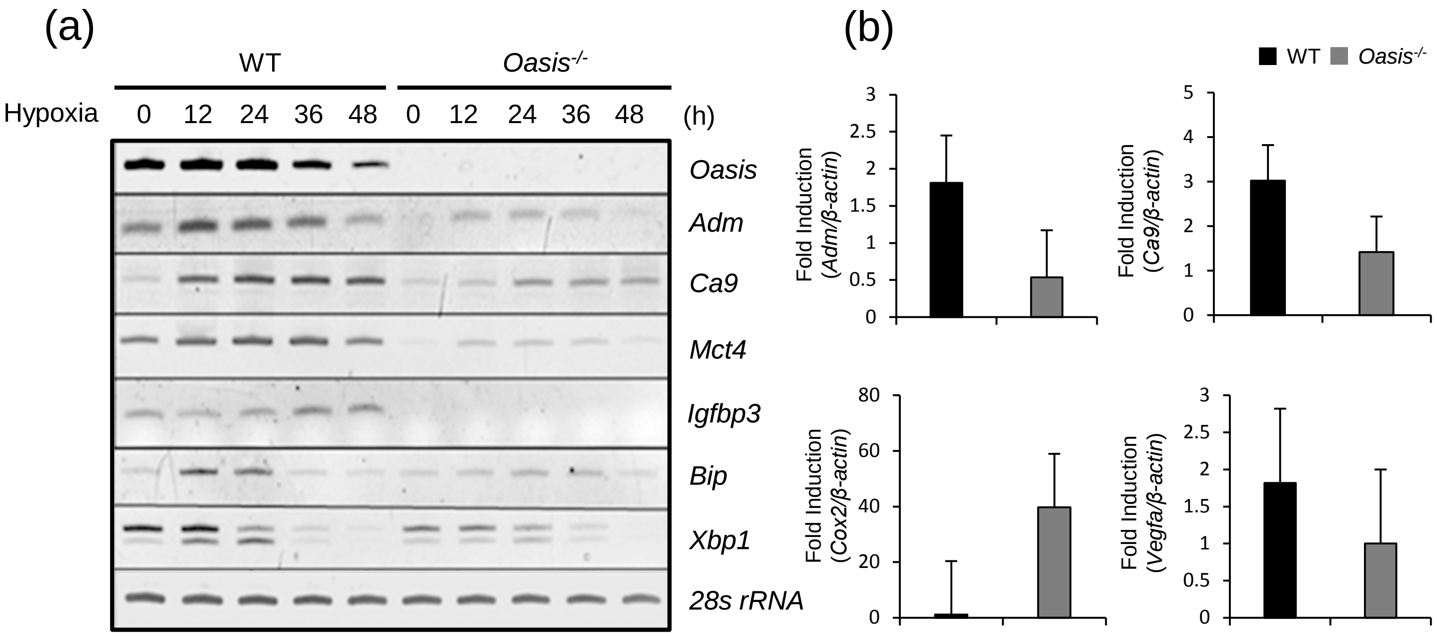

Supplementary Figure S1. OASIS regulates the expression of HIF-1α target genes.
(a) RT-PCR analyses of WT and *Oasis*$^{-/-}$ MEFs. Cells were exposed to hypoxia (0.2% oxygen) for the indicated time periods (mean ± s.d., n = 3; *P < 0.05, t-test). Note that the expression levels of the HIF-1α target genes are impaired as well as those of ER stress-related genes (*Bip* and *Xbp1*). (b) Real time-PCR analyses of WT and *Oasis*$^{-/-}$ MEFs in normal conditions. Relative expression levels of genes indicated in Fig. 1c at time 0.

Supplementary Figure S2

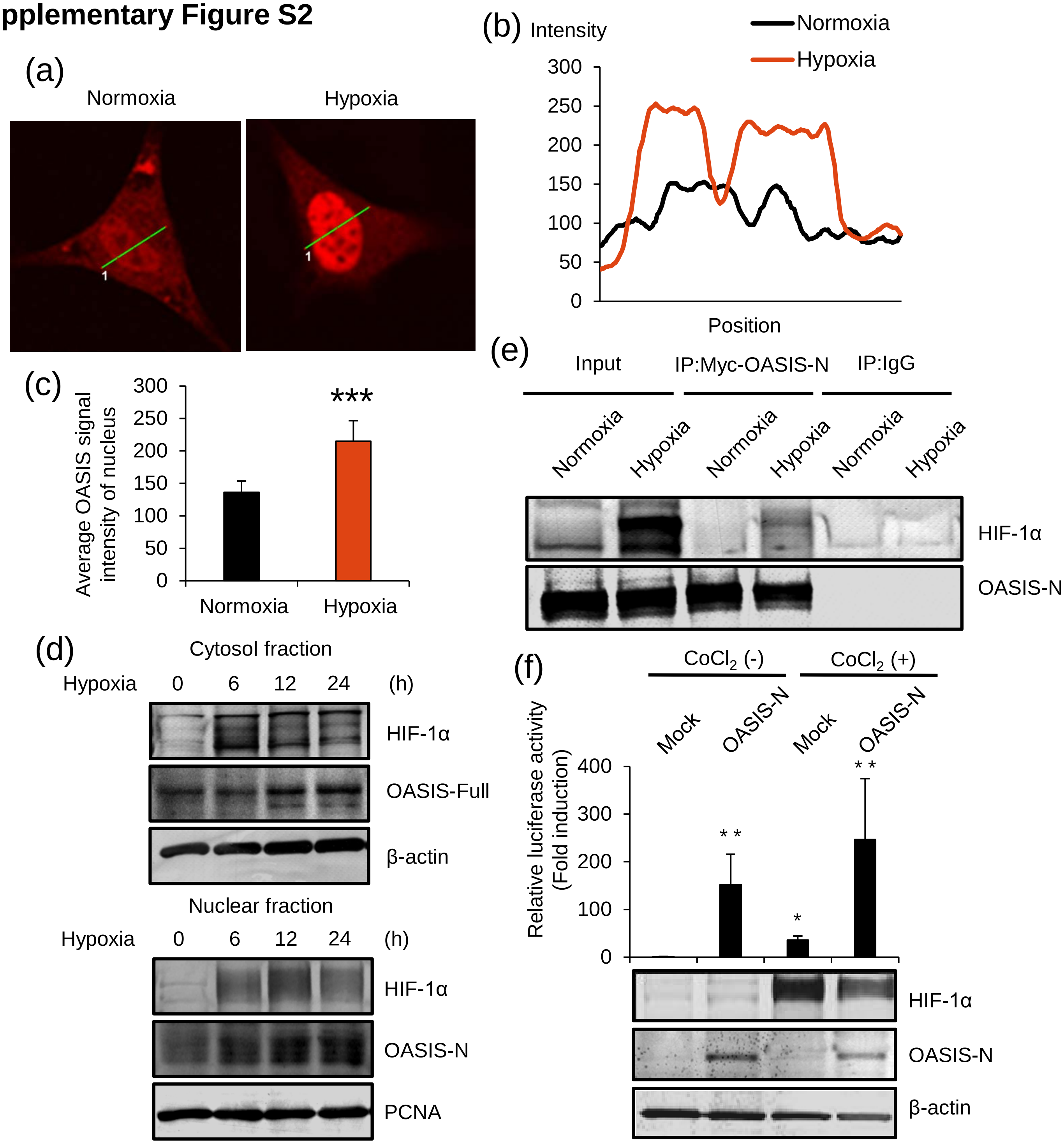

Supplementary Figure S2. OASIS is activated by hypoxia and interacts with HIF-1α.
(a, b, c) Quantification of immunofluorescence signal intensity. Intensities relative to the scale in the images were measured (187 positions on the scale were measured). Quantitation of every position is presented in panel b. Quantitation of the average nuclear signal is shown in panel c. Note that the signal intensity in the nucleus was increased by exposure to hypoxia (0.2% oxygen). (d) Western blot analyses of MC3T3-E1 cells in hypoxic conditions. Cells were exposed to hypoxia for indicated time periods. Cytosol and nuclear fractions were extracted and subjected to western blotting with anti-HIF-1α and anti-OASIS antibodies. Note that the levels of both OASIS-Full and OASIS-N were increased by hypoxia treatment. (e) Co-IP followed by western blot analyses of HIF-1α and OASIS. HEK293T cells were transfected with Myc-tagged OASIS-N and exposed to normoxia or hypoxia. Cell lysates were subjected to Co-IP assays with anti-Myc antibodies. The IP samples were subjected to western blotting with anti-HIF-1α and anti-OASIS antibodies. (f) Luciferase reporter analyses for the 5×HRE promoter in response to $CoCl_2$. HEK293T cells were cotransfected with pGL3-5×HRE and treated with 150 mM $CoCl_2$ or vehicle (mean ± s.d., n = 3; *P < 0.05, **P <0.01, t-test).

Supplementary Figure S3

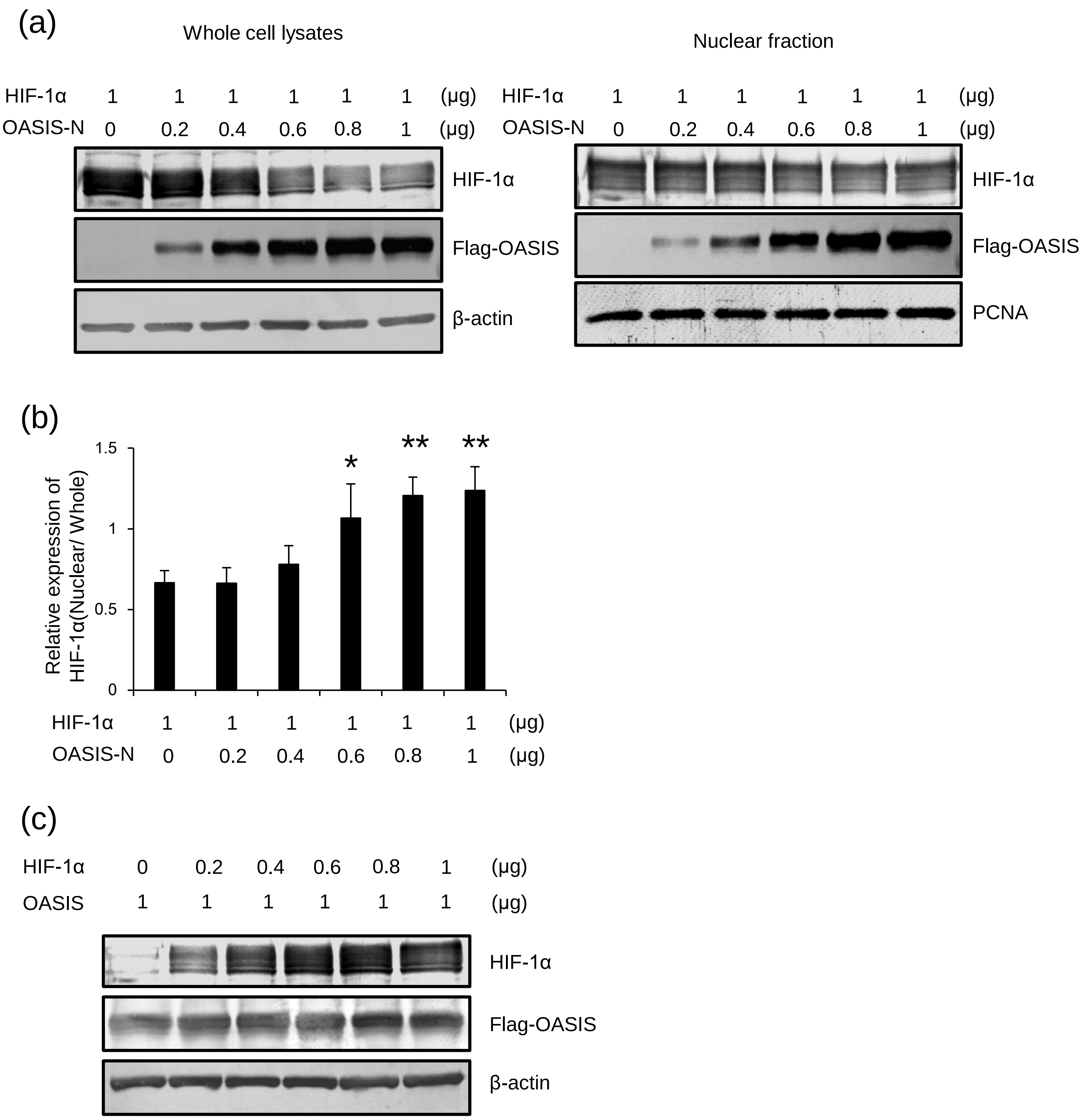

Supplementary Figure S3. OASIS is activated by hypoxia and interacts with HIF-1α.
(a, b) Western blot analyses of HIF-1α and OASIS. HEK293T cells were co-transfected with the indicated quantities of Flag-tagged OASIS-N and HIF-1α. Whole cell lysates and nuclear fractions were extracted and subjected to western blotting with anti-HIF-1α and anti-Flag antibodies. Note that an increased amount of OASIS leads to the down-regulation of HIF-1α expression in whole cell lysates (left) but not in the nucleus (right). Relative expression of nuclear HIF-1α is shown in panel b (mean ± s.d., n = 3; *P < 0.05, **P <0.01, t-test). (c) Western blot analyses of HIF-1α and OASIS. HEK293T cells were co-transfected with the indicated quantities of Flag-tagged OASIS-N and HIF-1α. Whole cell lysates subjected to western blotting with anti-HIF-1α and anti-Flag antibodies.

Supplementary Figure S4

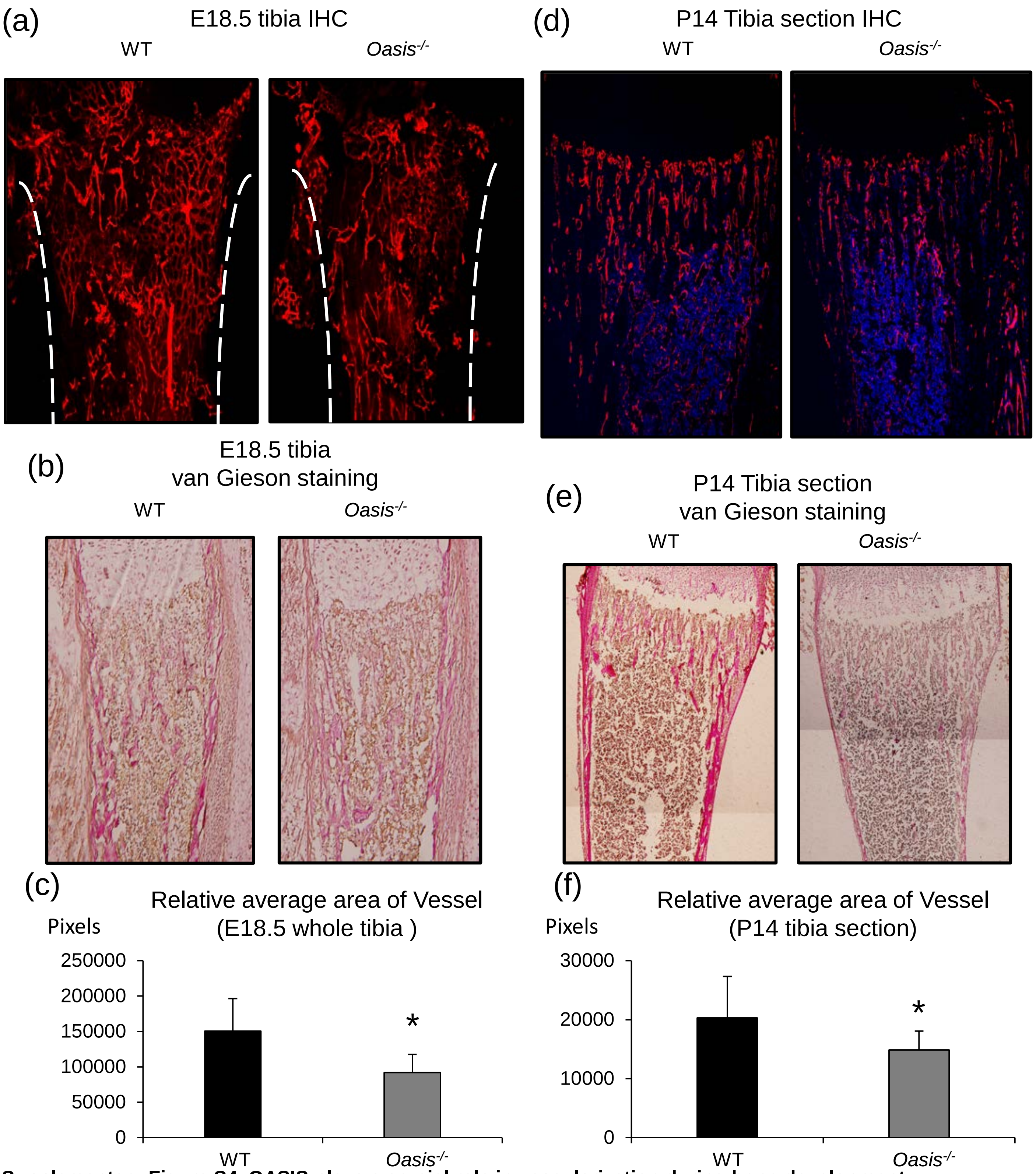

Supplementary Figure S4. OASIS plays a crucial role in vascularization during bone development.
(a, b, c) Immunofluorescence staining with anti-CD31 antibody in whole tibia (a) and Van Gieson staining of frozen tibial sections (b) from WT and *Oasis*$^{-/-}$ E18.5 mice. Note that decreased collagen matrix and retarded vascularization is observed in the *Oasis*$^{-/-}$ tibia. Quantitation of the vessel areas are indicated in panel c (mean ± s.d., *n* = 5; **P* <0.05, *t*-test). (d, e, f) Immunofluorescence staining with anti-CD31 antibody (d) and Van Gieson staining (e) of undecalcified frozen tibial sections from P14 WT and *Oasis*$^{-/-}$ mice. Note that decreased collagen matrix and retarded vascularization are observed in the *Oasis*$^{-/-}$ tibia. Quantitation of the vessel areas are indicated in panel f (mean ± s.d., *n* = 5; **P* <0.05, *t*-test).

Supplementary Figure S5

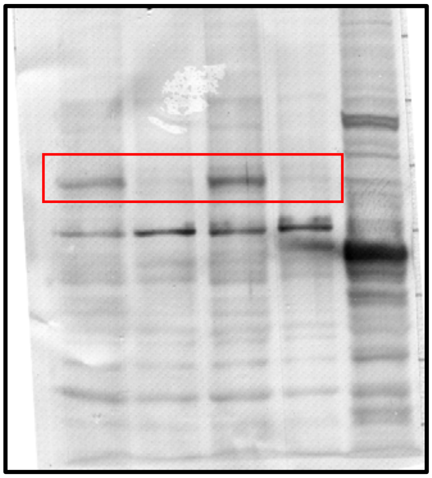

Fig. 1d middle (OASIS)

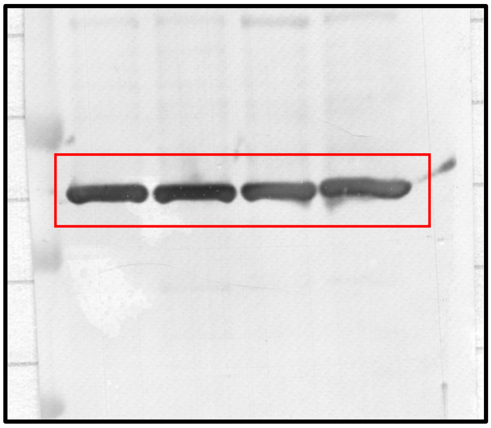

Fig. 1d bottom (β-actin)

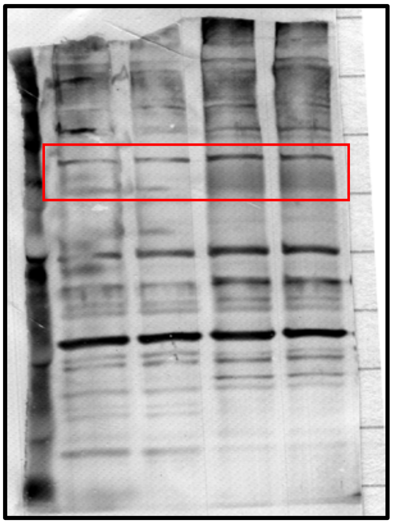

Fig. 1d top (HIF-1α)

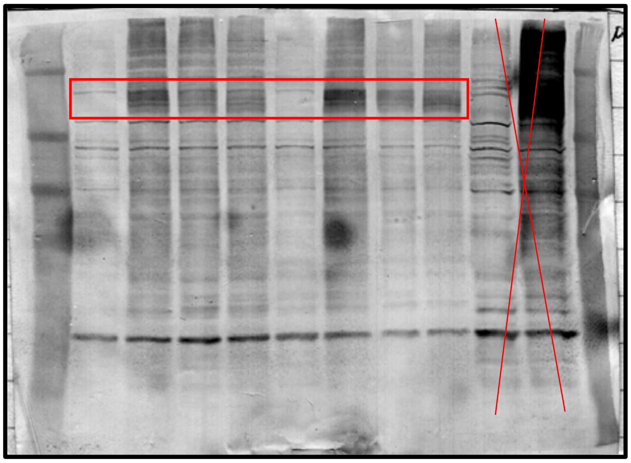

Fig. 1e top (HIF-1α)

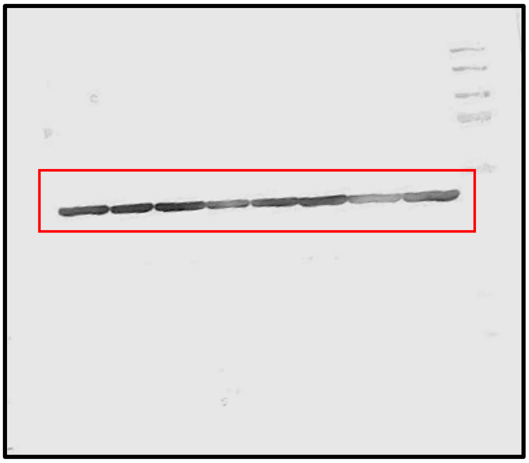

Fig. 1e bottom (β-actin)

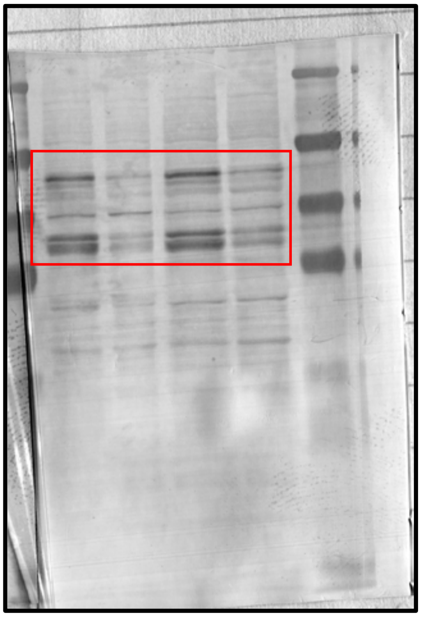

Fig. 1g top (HIF-1α)

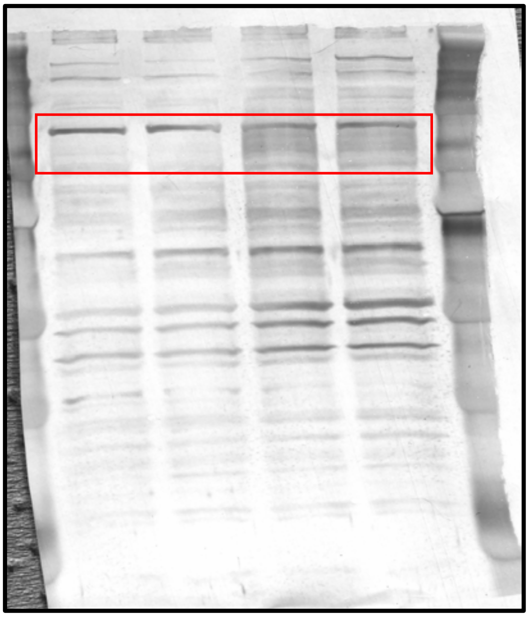

Fig. 1g middle (OASIS)

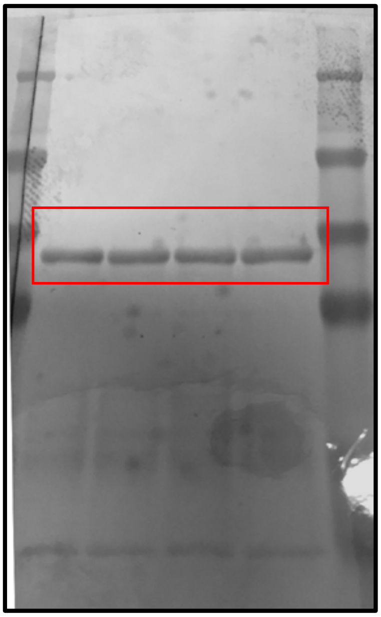

Fig. 1g bottom (β-actin)

Supplementary Figure S5. OASIS regulates the expression of HIF-1α target genes.
Full-length blots of Figure 1d, 1e and 1g to detect protein expressions of HIF-1α, OASIS and β-actin.

Supplementary Figure S6

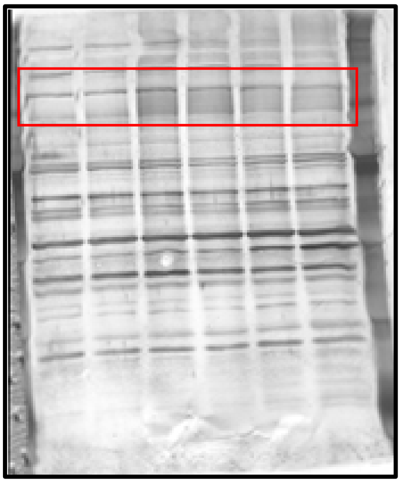

Fig. 2c top (HIF-1α)

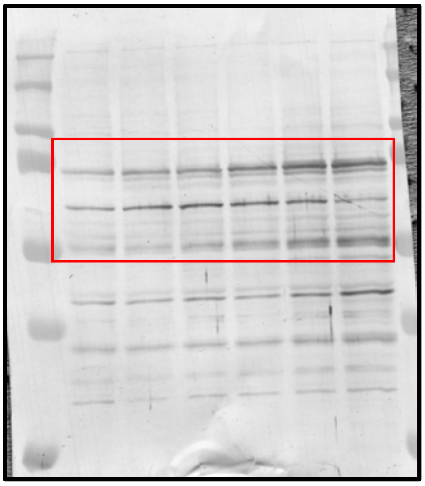

Fig. 2c middle (OASIS)

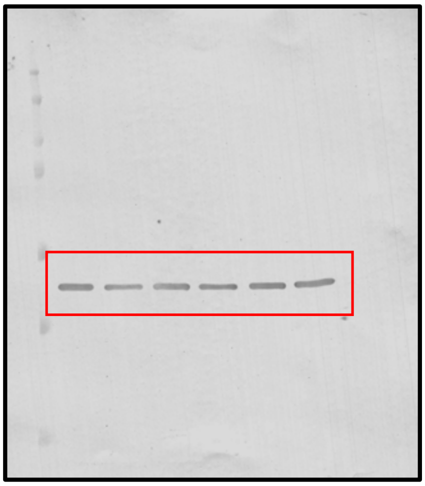

Fig. 2c bottom (β-actin)

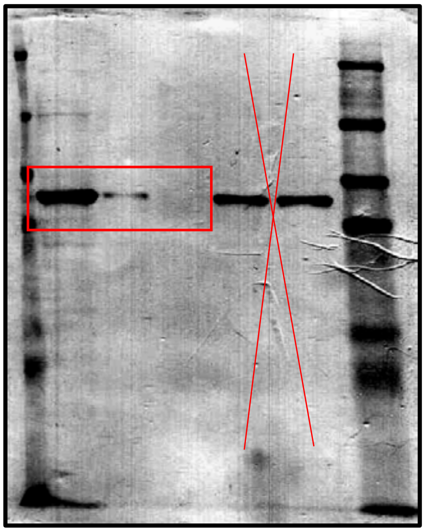

Fig. 2e left (OASIS)

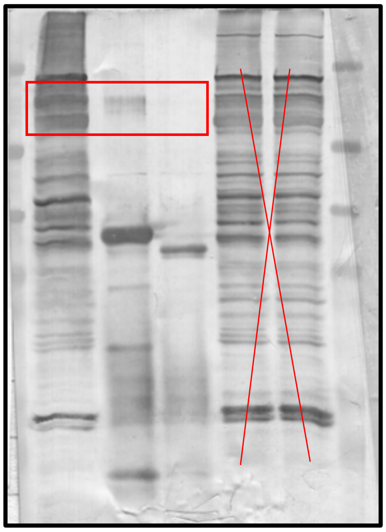

Fig. 2e left (HIF-1α)

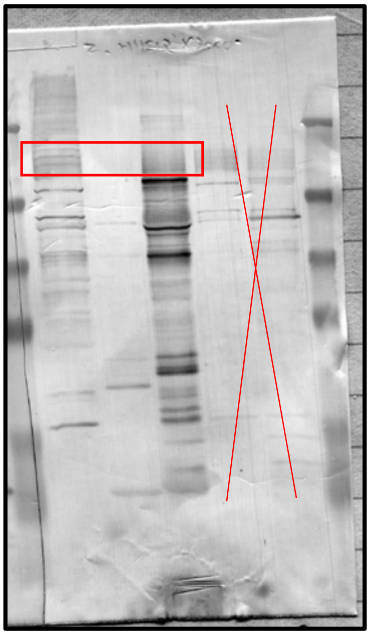

Fig. 2f top (HIF-1α)

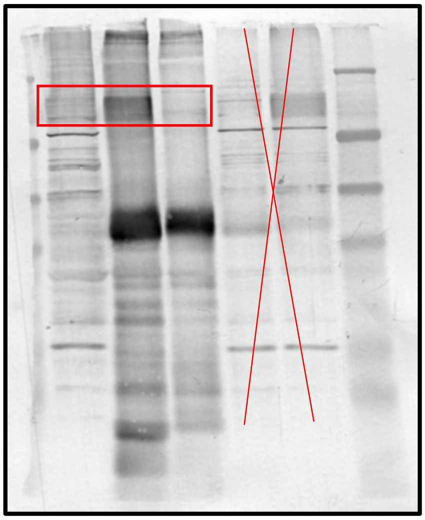

Fig. 2e right (HIF-1α)

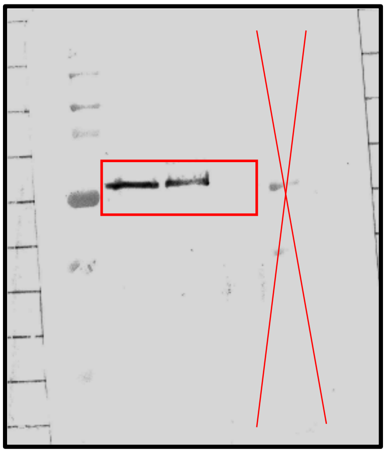

Fig. 2e right (OASIS)

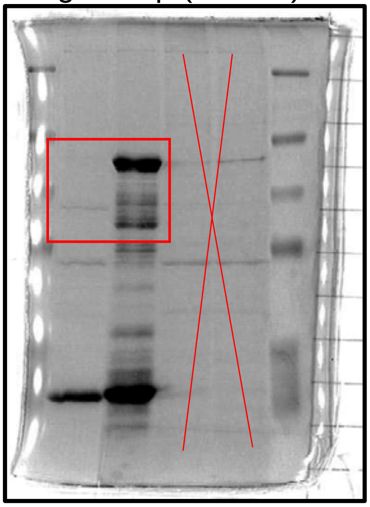

Fig. 2f bottom (CBB staining)

Supplementary Figure S6. OASIS is activated by hypoxia and interacts with HIF-1α.
Full-length blots of Figure 2c, 2e and 2f to detect protein expressions of HIF-1α, OASIS and β-actin.

Supplementary Figure S7

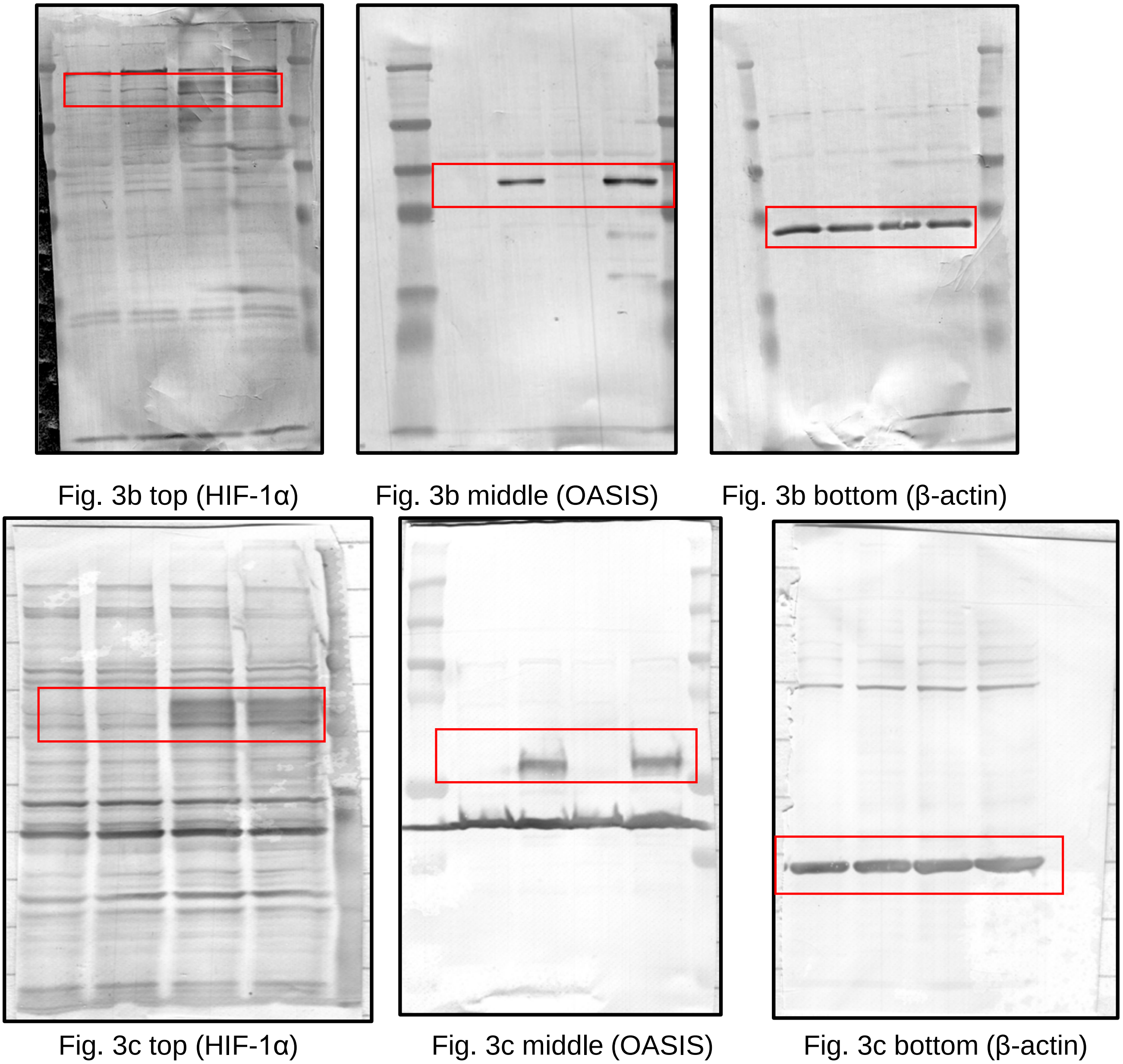

Supplementary Figure S7. OASIS enhances the promoter activity of the HRE.
Full-length blots of Figure 3b and 3c to detect protein expressions of HIF-1α, OASIS and β-actin.

Supplementary Figure S8

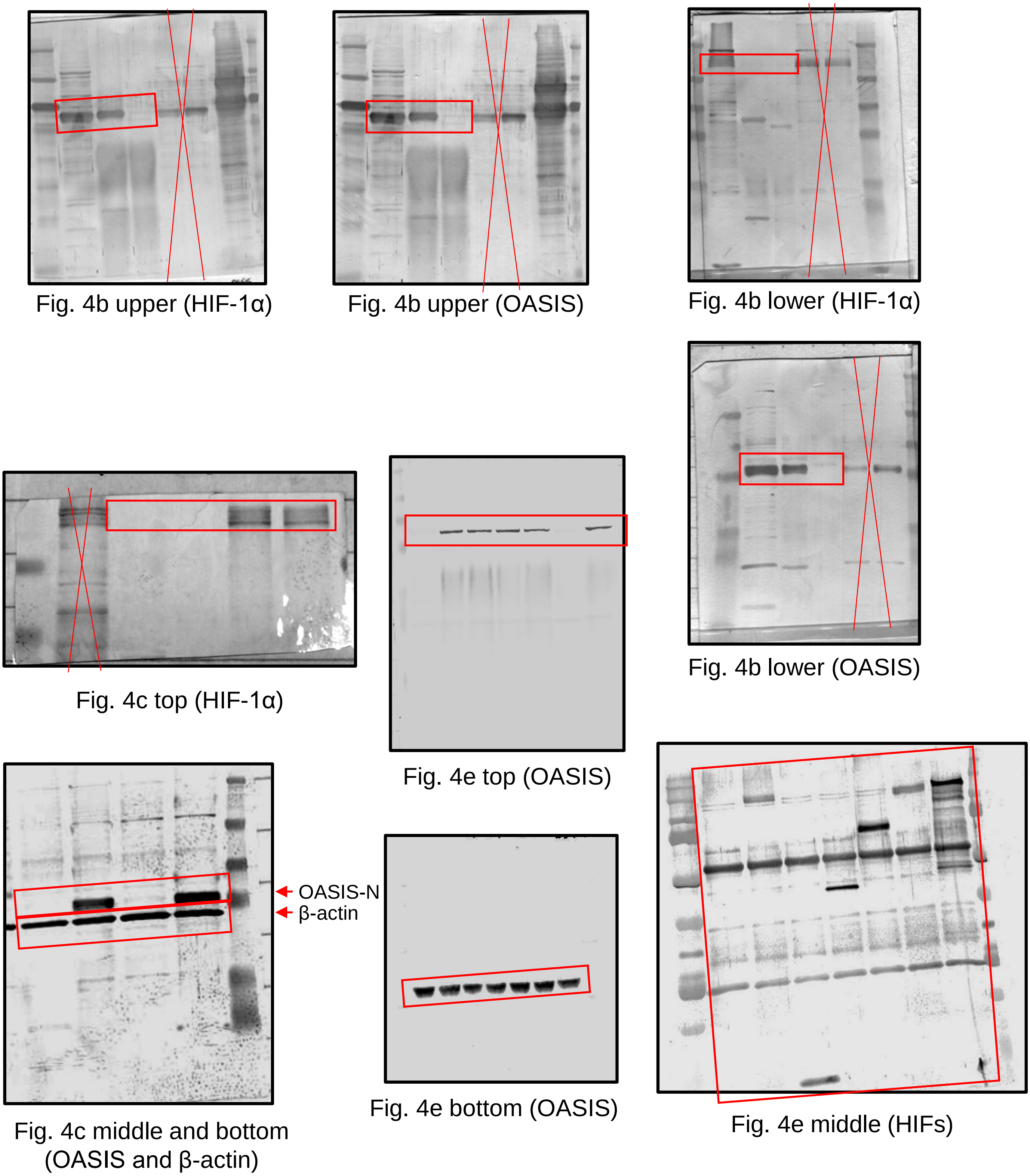

Supplementary Figure S8. bZIP domain of OASIS binds to the bHLH domain of HIFs.
Full-length blots of Figure 4b, 4c and 4e to detect protein expressions of HIFs, OASIS and β-actin.

Supplementary Table 1.

Primer list

ADM-Fwd	5'-CACCCTGATGTTATTGGGTTCA -3'
ADM-Rev	5'-TTAGCGCCCACTTATTCCACT-3'
Ca9-Fwd	5'-TGCTCCAAGTGTCTGCTCAG-3'
Ca9-Rev	5'-CAGGTGCATCCTCTTCACTGG-3'
Slc16a3(MCT4)-Fwd	5'-TCACGGGTTTCTCCTACGC-3'
Slc16a3(MCT4)-Rev	5'-GCCAAAGCGGTTCACACAC-3'
IGFBP3-Fwd	5'-CCAGGAAACATCAGTGAGTCC-3'
IGFBP3-Rev	5'-GGATGGAACTTGGAATCGGTCA-3'
PTGS2-Fwd	5'-CACCATTCTCCTTGAAAGGACTTATG-3'
PTGS2-Rev	5'-GCAGATGAGAGACTGAATTGAGGC-3'
beta-actin-Fwd	5'-TCCTCCCTGGAGAAGAGCTAC-3'
beta-actin-Rev	5'-TCCTGCTTGCTGATCCACAT-3'
BiP-Fwd	5'-GTTTGCTGAGGAAGACAAAAAGCTC-3'
BiP-Rev	5'-CACTTCCATAGAGTTTGCTGATAAT-3'
OASIS-Fwd	5'-CCTTGTGCCTGTCAAGATGGAG-3'
OASIS-Rev	5'-GCAGCAGCCATGGCAGAGGAG-3'
28s-F	5'-TTGAAAATCCGGGGGAGAG-3'
28s-R	5'-ACATTGTTCCAACATGCCAG-3'
VEGFA-Fwd	5'-AAGCTGGGTGAATGGAGCGA-3'
VEGFA-Rev	5'-CACGCACACACTCACTCA-3'
XBP1—Fwd	5'-ACACGCTTGGGAATGGACAC-3'
XBP1-Rev	5'-CCATGGGAAGATGTTCTGGG-3'

Supplementary Table 2.

Genes down-regulated by OASIS knockout

HIF1α target Genes	Wild type	*Oasis*$^{-/-}$	Fold change (*Oasis*$^{-/-}$/WT)
Adm	6.59	3.64	0.55
Bhlhb2	24.77	13.47	0.54
Ca9	5.73	1.35	0.24
Cox2	3.90	1.48	0.38
Mct4	6.27	1.42	0.23
Pfkfb3	2.76	1.58	0.57
Pgk1	163.5	65.58	0.40
Ppara	1.03	0.231	0.22
Vegfa	5.67	1.91	0.34

Fold changes of HIF-1α target genes from microarray analysis.
